# Reply to: Fundamental methodological issues

**DOI:** 10.1038/s41405-023-00159-3

**Published:** 2023-07-24

**Authors:** Bennett Tochukwu Amaechi, Thais Santiago Phillips, Betty Isabel Perozo, Yuko Kataoka, Fatemeh Movaghari Pour, Rayane Farah, Amos Chinedu Obiefuna, Moshtagh Rashid Farokhi

**Affiliations:** 1 Department of Comprehensive Dentistry, School of Dentistry, University of Texas Health San Antonio, 7703 Floyd Curl Drive, San Antonio, TX 78229-3900 USA; 2 ANCOSTAT Statistical Consulting, San Antonio, TX 78245 USA

**Keywords:** Dentistry, Health care

Thank you very much for giving us an opportunity to publish our work [1] in this high quality and internationally renowned peer-reviewed journal, *BDJ Open*, and for allowing us to respond to the letter from Dr Chris Longbottom.

In acknowledging Dr Longbottom’s response to our paper we are, of course, cognisant of his expertise in this field and his role as a consultant to a company that developed a technology aimed at performing the same function as our investigated technology (differentiating between active and inactive caries lesions). We understand his point that using a single examiner to conduct both the ICDAS examination and LumiCare assessment may have compromised the blinding as well as predisposed the study to the risk of bias. However, we wish to emphasise that this was a pilot and a proof-of-concept study, and that we are presently conducting a large multicentre clinical trial in which we have rectified this potential shortcoming.

With regards to proximity in time of the ICDAS and LC rinse examinations, we must emphasise that anyone who is familiar with conducting a full mouth ICDAS examination, simultaneously differentiating between active, inactive and hypomineralisation, would appreciate the difficulty (almost impossibility), after 20 min of ICDAS examination, to recognise exactly how they graded each lesion (active, inactive or hypomineralisation) by simply looking at fluorescent and nonfluorescent lesions. We are therefore content that the risk of bias in this case is extremely low. With regards to the difference in the sensitivity and Odds Ratio figures of the ‘LC Rinse’ assessment for the ‘Selected teeth’ and ‘Full dentition’ surfaces, the lesions in the selected study teeth in each subject were diagnosed and selected by an expert in caries diagnosis and detection (who is not the calibrated clinical examiner), particularly to determine the LC rinse ‘assessment window’. Unfortunately, we were not able to detail this in the publication due to the word limitation.

It is pertinent to mention that our study was not designed to measure and compare the area of the lesion detected by ICDAS examination to area of the corresponding lesion detected by LC rinse assessment. *Figure 1* obviously shows the qualitative distinction between the green fluorescence of the caries lesions and yellow fluorescence of the rest of the tooth surface. The ICDAS-detected lesions in *Figure 1a* run along the gingival crevice (typical plaque stagnation and caries prone area) just as the LC Rinse-detected fluorescent areas (*Figure 1b*) corresponding to the ICDAS-detected lesions run along the gingival crevice, so it is absolutely wrong for one to claim that the green fluorescence of the illuminated lesions came from the gingival crevice.

Although we collected data on the distribution figures for the occlusal and free smooth surface sites, due to space limitation we could not show this distribution in our publication. Consequently, it is inappropriate for a reader to guess that the distribution may have influenced the study outcomes. Nevertheless, we do believe that our published study demonstrated the efficacy of the LC Rinse in distinguishing between active caries, inactive caries and hypomineralisation, and therefore can augment caries detection.
